# Mutated Flt3Lg Provides Reduced Flt3 Recycling Compared to Wild-Type Flt3Lg and Retains the Specificity of Flt3Lg-Based CAR T-Cell Targeting in AML Models

**DOI:** 10.3390/ijms24087626

**Published:** 2023-04-21

**Authors:** Varvara Maiorova, Murad D. Mollaev, Polina Vikhreva, Dmitriy M. Chudakov, Alexey Kibardin, Michael A. Maschan, Sergey Larin

**Affiliations:** 1 Dmitriy Rogachev National Medical Center of Pediatric Hematology, Oncology and Immunology, 117997 Moscow, Russia; 2 Center of Life Sciences, Skolkovo Institute of Science and Technology, 121205 Moscow, Russia; chudakovdm@gmail.com; 3 Institute of Translational Medicine, Pirogov Russian National Research Medical University, 117997 Moscow, Russia

**Keywords:** ligand-based targeting, acute myeloid leukemia, natural-ligand chimeric antigen receptor (CAR) T, fms-like tyrosine kinase 3 (Flt3), fms-like tyrosine kinase 3 ligand variant (Flt3Lg-L27P) bioactivity

## Abstract

The cells of acute myeloid leukemia are defined by clonal growth and heterogenous immunophenotypes. Chimeric antigen receptors (CARs) commonly recognize molecular targets by single-chain antibody fragments (scFvs) specific to a tumor-associated antigen. However, ScFvs may form aggregates, thus stimulating tonic CAR T-cell activation and reducing CAR T-cell functioning in vivo. Harnessing natural ligands as recognition parts of CARs, specific targeting of membrane receptors can be achieved. Previously, we presented ligand-based Flt3-CAR T-cells targeting the Flt3 receptor. The extracellular part of Flt3-CAR consisted of full-size Flt3Lg. Meanwhile, upon recognition, Flt3-CAR may potentially activate Flt3, triggering proliferative signaling in blast cells. Moreover, the long-lasting presence of Flt3Lg may lead to Flt3 downregulation. In this paper, we present mutated Flt3Lg-based Flt3m-CAR (‘m’—for ‘mutant’) T-cells targeting Flt3. The extracellular part of Flt3m-CAR consists of full-length Flt3Lg-L27P. We have determined that ED_50_ for recombinant Flt3Lg-L27P produced in CHO cells is at least 10-fold higher than for the wild-type Flt3Lg. We show that the mutation in the recognizing domain of Flt3m-CAR did not affect the specificity of Flt3m-CAR T-cells when compared to Flt3-CAR T-cells. Flt3m-CAR T-cells combine the specificity of ligand–receptor recognition with reduced Flt3Lg-L27P bioactivity, leading to potentially safer immunotherapy.

## 1. Introduction

Despite huge advances in immunotherapy research and numerous applications, acute myeloid leukemia (AML) treatment protocols are still based on intensive chemotherapy, bearing significant side effects. Chemotherapy is used to eliminate blast cells and prepare the patient for hematopoietic stem cell transplantation (HSCT). However, leukemic cells can become resistant to chemotherapy, leading to a relapse. Chimeric antigen receptor (CAR) T-cell therapy is proposed as a viable alternative to traditional chemotherapy, being a more specific and less toxic approach to cytoreduction.

The clonal heterogeneity of AML and the associated heterogeneity of surface antigen expression is an important barrier to successful CAR-T therapy in AML. Each clone of leukemic cells may bear a distinct immunophenotype. Moreover, AML blasts’ immunophenotype may vary significantly between patients. Targeting a single AML-associated antigen could lead to an antigen-negative relapse similar to the CD19-negative relapses (CD—cluster of differentiation) during B-cell acute lymphoblastic leukemia treatment with CD19 targeting [1]. Simultaneous targeting of different AML-associated antigens in compliance with particular blast cells’ immunophenotypes may be a promising approach. A variety of antigens are being tested to target AML blast cells, including CD7, CD19, CD33, CD38, CD44v6, CD70, CD123, CD276, CLL-1, Flt3, and NKG2D [2].

Recently, five CAR T-cell therapies for treating hematological diseases were approved [3,4,5,6,7,8]. Four of them target B-cell antigen CD19. Moreover, all four CAR constructs contain single-chain variable fragments (scFv) originating from the mouse monoclonal antibody FMC63 sequence [9]. Expanding the success of the CAR T-cell approach for B-cell malignancies to AML requires designing and thoroughly studying new scFv fragments specific to AML-associated antigens.

The triumph of FMC63-based chimeric receptors is associated with the fact that anti-CD19 CARs are less prone to aggregate on the CAR T-cell surface [10]. In a monoclonal antibody molecule, the amino acid residues of the constant region commonly shield the hydrophobic residues of a variable region [11]. Deprived of the constant region, single chain variable fragments (scFvs) often form aggregates [12]. Aggregate-forming scFvs stimulate tonic activation of CAR T-cells [13]. The tendency to aggregate is a characteristic feature of a particular CAR genetic construct. For instance, when the framework region of scFvs in the GD2-CAR were replaced with the corresponding region of the CD19-CAR, tonic activation of GD2-CAR T-cells was reduced [10]. Tonic activation leads to decreased antitumor efficacy due to fast CAR T-cell exhaustion, as well as a lack of persistence [14,15]. When designing a new CAR sequence, the pH- and temperature-dependent solubility of chimeric protein should be carefully studied for each new scFv.

In the quest for an alternative to antibody fragments, a number of studies have focused on natural ligand- and receptor-based CAR receptors targeting tumor antigens [16]. This approach allows for the use of a large body of evidence on ligand–receptor binding affinities, functional domains, and functional mutations during CAR sequence design. Point mutations may reduce or increase binding affinity for the target molecule. For instance, IL13-E13Y-containing CARs showed a 50-fold higher affinity for IL13Ra2 and a 5-fold lower affinity for IL13Ra1 than wild-type IL13 in glioblastoma multiform models [16]. 

Previously, we presented ligand-based Flt3-CAR T-cells specifically targeting the Flt3 receptor [17]. Flt3 is a tyrosine kinase receptor, which is normally present on hematopoietic progenitor cells and dendritic cells. Upregulated in most AML cases, the Flt3 receptor represents an attractive molecular target for immunotherapy. Flt3Lg, an agonist of Flt3, forms a noncovalent dimer. However, dimeric Flt3Lg binds to the Flt3 receptor, forming a high-affinity homodimeric receptor complex that triggers intracellular proliferative signaling, thus acting as an undesirable tumor growth factor. The L27 position was described to belong to the Flt3Lg dimerization interface. L27 mutation leads to both a significant decrease in the biological activity of Flt3Lg [18] and changes in the 3D structure of the Flt3Lg dimer [19].

The present work is the first to the best of our knowledge that presents the Flt3Lg-L27P-based Flt3m-CAR T-cells. The extracellular recognition part of the Flt3m-CAR receptor consisted of full-length Flt3Lg-L27P. In vitro models demonstrated that L27P mutation did not affect the specificity of the Flt3m-CAR T-cell-mediated killing. We show that recombinant Flt3Lg-L27P does not interfere with the Flt3m-CAR T-cell-mediated killing despite the significantly reduced bioactivity of recombinant Flt3Lg-L27P as compared to the wild-type Flt3Lg. We propose that the reduced specific activity of the natural ligand-based domain of Flt3m-CAR should limit any possible interference with endogenous Flt3Lg signaling, suggesting the basis for safer immunotherapy.

## 2. Results

### 2.1. Recombinant Flt3Lg-L27P Did Not Disrupt Wild-Type Flt3Lg Bioactivity

Recombinant Flt3Lg-L27P, produced in yeasts, was previously presented as a monomer form of Flt3Lg [18]. Recombinant Flt3Lg-L27P preserved binding to the Flt3 receptor while its specific activity was significantly reduced.

**Figure 1:**
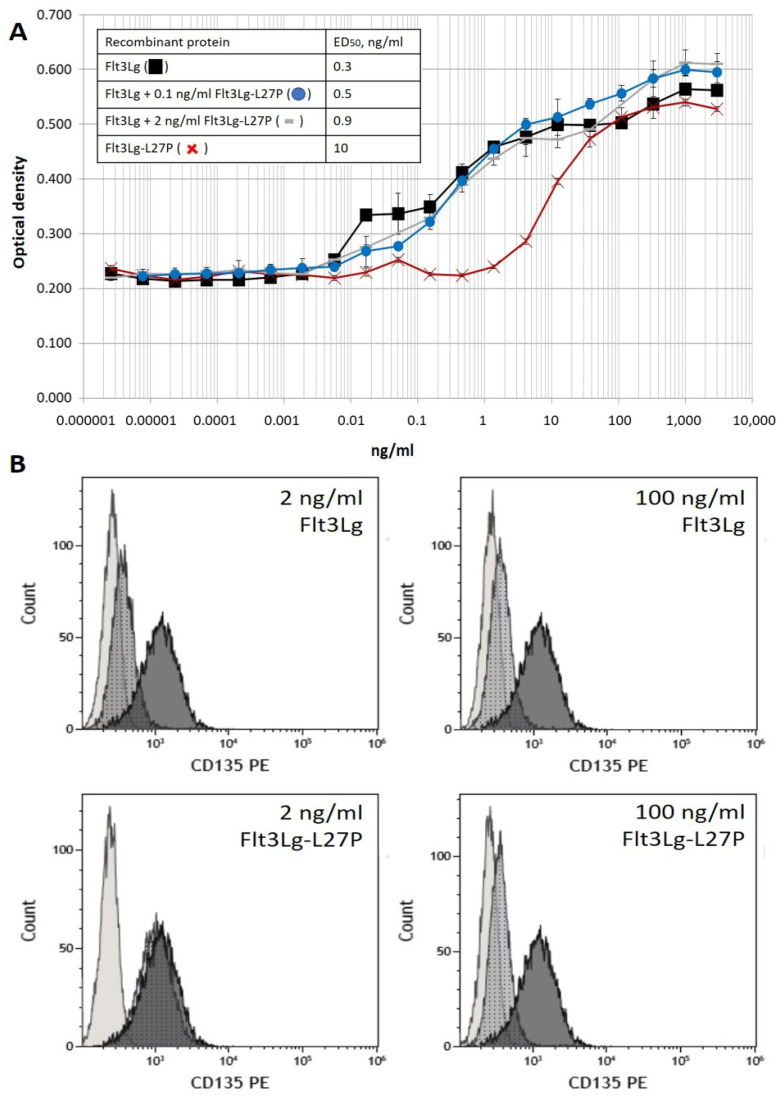
(**A**) Measuring ED_50_ for recombinant Flt3Lg-L27P and Flt3Lg via induced proliferation in the THP-1 cell line. Stimulated proliferation was assessed in the MTS assay. ED_50_ concentrations were determined using the mean OD values of the lower and upper plateaus. Black squares correspond to wild-type Flt3Lg and red crosses to Flt3Lg-L27P. Wild-type Flt3Lg-stimulated THP-1 proliferation in the presence of 0.1 ng/mL (blue circles) or 2 ng/mL (gray dashes) was also assessed. Data were represented as mean ± SD. ED_50_ values are presented in the figure. (**B**) Stimulation of Flt3 downregulation in the THP-1 cell line using recombinant Flt3Lg and Flt3Lg-L27P. THP-1 cells were incubated in the presence of 2 or 100 ng/mL of either Flt3Lg (upper pictures) or Flt3Lg-L27P (lower pictures) for 18 h. The expression of Flt3 in THP-1 cells was detected using flow cytometry after staining the cells with an anti-CD135-PE antibody. For all pictures, dark grey histograms correspond to non-stimulated THP-1 stained with anti-CD135-PE, light-grey histograms correspond to non-stained THP-1 cells, and dotted histograms correspond to stimulated THP-1 cells stained with anti-CD135-PE.

In this work, human recombinant protein Flt3Lg-L27P was purified from the mammalian producing cells medium. Previously, we showed that soluble recombinant Flt3Lg stimulated THP-1 cell proliferation [17]. Briefly, using an optimized MTS assay, we were able to determine a half-effective dose (ED_50_) of cytokine. Similarly, ED_50_ was measured for recombinant Flt3Lg-L27P (Figure 1A). ED_50_ of recombinant Flt3Lg-L27P was more than 10-fold higher than that of wild-type Flt3Lg (Figure 1A). The bioactivity of wild-type Flt3Lg in the presence of Flt3Lg-L27P was also determined. For both cytokines, the 0.1 ng/mL concentration is below ED_50_; at 2 ng/mL, Flt3-L27P has little proliferative activity, while for the wild-type Flt3 ligand, this concentration is 10-fold higher than ED_50_. Interestingly, the presence of Flt3Lg-L27P at the mentioned concentrations in the incubation medium did not alter the bioactivity of wild-type Flt3Lg.

### 2.2. Low Doses of Recombinant Flt3Lg-L27P Did Not Stimulate Flt3 Downregulation, While Flt3Lg Did

Prolonged exposure of THP-1 cells to Flt3Lg was associated with Flt3 receptor downregulation in THP-1 cells [20]. If the effect correlates with Flt3Lg bioactivity, then the mutated Flt3Lg-L27P with reduced bioactivity should less influence the Flt3 receptor surface presence. According to the proliferative curve, mutated Flt3Lg-L27P was not active at the 2 ng/mL concentration, but ED_50_ for the 100 ng/mL concentration was 10-fold higher than ED_50_ for Flt3Lg-L27P. For wild-type Flt3Lg, both concentrations exceeded ED_50_ (Figure 1A). We proposed that lower concentrations of Flt3Lg-L27P should not lead to Flt3 receptor downregulation.

To prove this statement, we incubated Flt3-positive THP-1 cells in the presence of 2 ng/mL or 100 ng/mL of Flt3Lg or similar concentrations of Flt3Lg-L27P for 18 h. Subsequently, to measure the presence of the Flt3 receptor, the cells were stained with anti-Flt3 PE. Both concentrations of Flt3Lg led to Flt3 downregulation, while the presence of 2 ng/mL of Flt3Lg-L27P had little effect on the presence of Flt3 on the surface of THP-1 cells (Figure 1B). Additionally, a control of 100 ng/mL of Flt3Lg was added to intact THP-1 cells during the staining procedure to prove that anti-Flt3 PE would not interfere with Flt3Lg:Flt3 interaction (Figure S1).

Flt3 receptor downregulation through interaction with soluble Flt3Lg may become one of the ways for blast cells to escape Flt3-CAR T-cell-mediated killing. Using the Flt3Lg-L27P sequence as the recognizing domain, the Flt3m-CAR (‘m’—for ‘mutant’) receptor could potentially resolve this issue.

### 2.3. Generating Flt3m-CAR T-Cells

**Figure 2:**
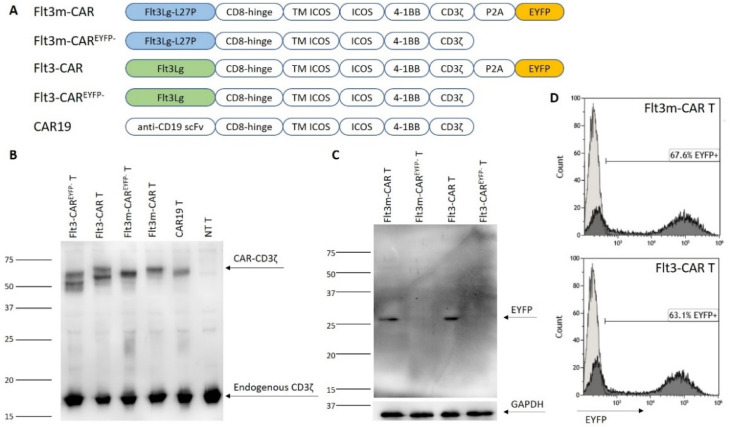
Generating Flt3-CAR T-cells. (**A**) Schematic structure of Flt3m-CAR, Flt3m-CAR^EYFP−^, Flt3-CAR, Flt3-CAR^EYFP−^, and CAR19 coding regions. (**B**) In the Western blot images, the synthesis of chimeric antigen receptors was demonstrated. The bands corresponding to the CD3ζ chain that belongs to CAR receptors appeared in the lines with CAR T-cell lysates (50–60 kDa), but not in the line with non-transduced (NT) T-cell lysates. Bands corresponding to the CD3ζ chain as part of endogenous T-cell receptors (16 kDa) are present in all lines. (**C**) Using the Western blot technique, the synthesis and proper cleavage of the EYFP protein in Flt3m-CAR T-cells was shown. Arrows indicate a single band of an appropriate size of EYFP (27 kDa). (**D**) EYFP synthesis in Flt3m-CAR T-cells was assessed on the seventh day after transduction using flow cytometry. A representative figure is shown. The experiments were performed at least three times.

In this work, an Flt3m-CAR construct was designed (Figure 2A). The recognizing part of the coding region consists of a full-length Flt3Lg sequence bearing an L27P mutation in the Flt3Lg dimerization interface. Activating domains were designed similarly to the Flt3-CAR construct. Briefly, the Flt3Lg-L27P coding region was connected via a CD8 hinge to the co-stimulatory domain (ICOS and 4-1BB) coding regions and to the activating domain (CD3ζ) coding region. Through a self-cleaving P2A sequence at the end of the construct, the EYFP coding sequence was added for easier detection of CARs. The control construct Flt3m-CAR^EYFP−^ lacked the EYFP sequence. Flt3-CAR, Flt3-CAR^EYFP−^, and CAR19 constructs were designed as described previously [17].

Donor T-cells were lentivirally transduced with the constructs bearing viral particles. Flt3m-CAR and Flt3-CAR^EYFP−^ protein biosynthesis was confirmed via Western blotting of the CAR T-cell lysates with anti-CD3ζ chain staining (Figure 2B). Lanes 3 and 4 were loaded with Flt3m-CAR^EYFP−^ T-cell and Flt3m-CAR T-cell lysates, respectively. The two left lanes were loaded with Flt3-CAR^EYFP−^ T-cell and Flt3-CAR T-cell lysates, and lanes 5 and 6 were loaded with CAR19 T-cell and non-transduced (NT) T-cell lysates. For all the transduced CAR T-cell lanes, the CD3ζ chain was visible in the 50–65 kDa region, corresponding to the intracellular part of chimeric receptors. The endogenous CD3ζ chain was detected in all lanes in the 16 kDa region.

Cleavage of the EYFP protein in Flt3m-CAR T-cells was also confirmed by Western blotting with anti-EYFP staining (Figure 2C). The bands related to the EYFP protein were located in the 26 kDa region for lanes 1 and 3, related to Flt3m-CAR T-cell and Flt3-CAR T-cell lysates, respectively. No residual fused proteins were detected within the region of 75–90 kDa.

Similarly to Flt3-CAR T-cells, EYFP-positive cells were referred to as Flt3m-CAR-positive cells (Figure 2D). Transduction efficiency for Flt3m-CAR lentivirus (67.6%) was comparable to that of Flt3-CAR (63.1%).

### 2.4. Flt3m-CAR T-Cells Showed Cytotoxicity Similar to That of Flt3-CAR T-Cells

**Figure 3:**
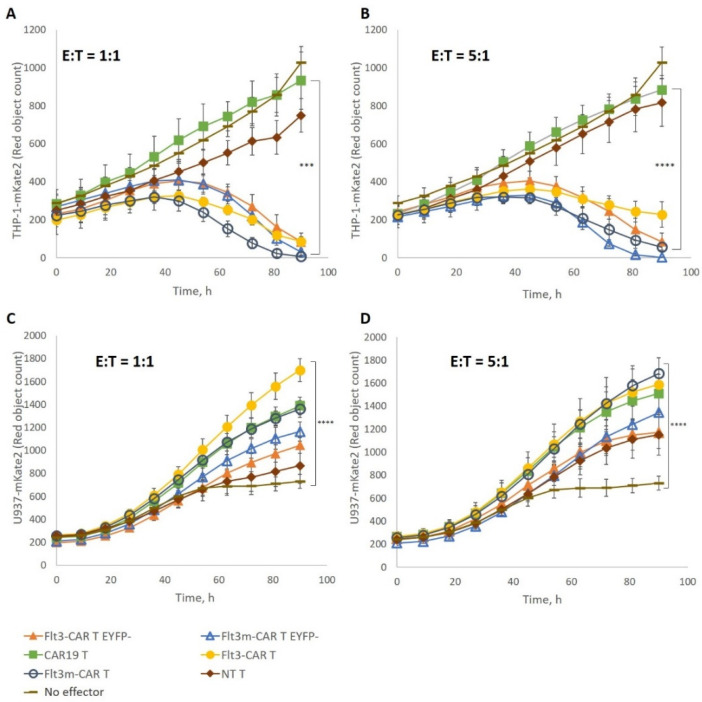
Specific Flt3m-CAR T-cell-mediated killing of target cells. Flt3m-CAR T-cells, Flt3m-CAR^EYFP−^ T-cells, Flt3-CAR T-cells, Flt3-CAR^EYFP−^ T-cells, CAR19 T-cells, and non-transduced (NT) T-cells were co-incubated with THP-1-mKate2 cells at an E:T ratio of 1:1 (**A**) or 5:1 (**B**) or with U937-mKate2 cells at an E:T ratio of 1:1 (**C**) or 5:1 (**D**). Empty dark blue circles refer to Flt3m-CAR T-cells, empty blue triangles to Flt3m-CAR^EYFP−^ T-cells, yellow circles to Flt3-CAR T-cells, orange triangles to Flt3-CAR^EYFP−^ T-cells, green squares to CAR19 T-cells, brown diamonds to NT T-cells, and dashes to wells with THP-1-mKate2 or U937-mKate2 cells without any effector cells. Red object counts are shown in the figure, indicating the number of alive THP-1-mKate2 or U937-mKate2 cells. Data are represented as mean ± SD. Mann–Whitney U-tests and Kruskal–Wallis tests were performed to identify significant differences. *** *p* < 0.001; **** *p* < 0.0001.

We used two tumor cell lines, Flt3-positive THP-1-mKate2 and Flt3-negative U937-mKate2, to test the cytotoxicity and specificity of Flt3m-CAR T-cells. Flt3m-CAR T-cells were incubated with Flt3-positive THP-1-mKate2 cells at an E:T ratio of 1:1 or 5:1 for 4 days (Figure 3A,B). For both E:T ratios, Flt3m-CAR T-cells eliminated Flt3-positive cells as efficiently as Flt3-CAR T-cells. Similarly, Flt3m-CAR T-cells were incubated with Flt3-negative U937-mKate2 cells at an E:T ratio of 1:1 or 5:1 (Figure 3C,D). Flt3m-CAR T-cells did not interfere with the proliferation of Flt3-negative U937-mKate2 cells at either E:T ratio. The L27P mutation of the Flt3Lg sequence in the Flt3m-CAR receptor did not affect the cytotoxicity of the chimeric receptor against Flt3-positive THP-1-mKate2 cells. Flt3-negative U937-mKate2 cells’ proliferation was not disrupted in the presence of Flt3m-CAR T-cells.

In two experiments, we tested if Flt3m-CAR^EYFP−^ T-cells exposed to target cells specifically secrete IFNγ and TNFα using ELISA. After 24 h of incubation with Flt3-positive THP-1 cells, Flt3m-CAR^EYFP−^ T-cells secreted (243 ± 127) pg/mL IFNγ and (47 ± 2) pg/mL TNFα. In the experiment with Flt3-negative U937 as target cells, Flt3m-CAR^EYFP−^ T-cells secreted (62 ± 19) pg/mL IFNγ. Flt3m-CAR^EYFP−^ T-cells without any target cells secreted (51 ± 20) pg/mL IFNγ. The level of TNFα was below detection. Thus, Flt3m-CAR^EYFP−^ T-cells specifically secreted IFNγ and TNFα when exposed to Flt3-positive THP-1. The TNFα level when Flt3m-CAR^EYFP−^ T-cells were in the presence of Flt3-negative U937 or without any target was similar (Figure S3).

**Figure 4:**
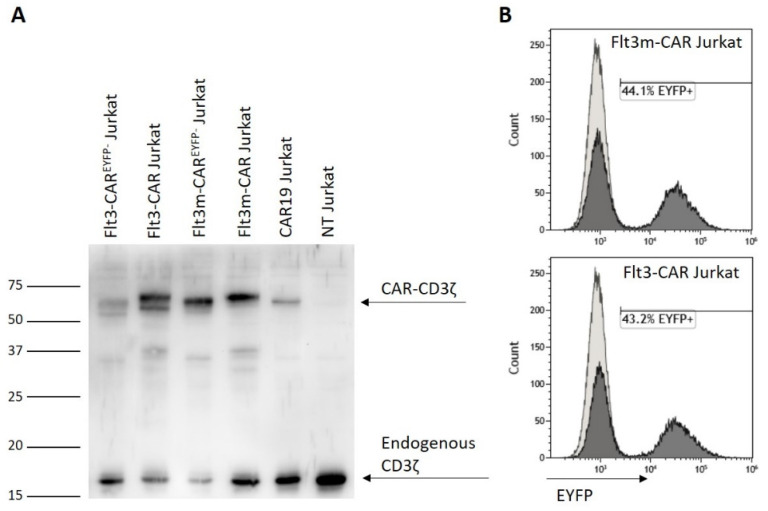
Generating Flt3m-CAR Jurkat cells. (**A**) In Western blot images, the synthesis of chimeric antigen receptors was demonstrated. The bands corresponding to the CD3ζ chain that belongs to CAR receptors appeared in the lines with CAR Jurkat cell lysates (50–60 kDa), but not in the line with non-transduced (NT) Jurkat cell lysates. The bands corresponding to the CD3ζ chain as part of endogenous T-cell receptors (16 kDa) are present in all lines. (**B**) EYFP synthesis in Flt3m-CAR Jurkat cells was assessed on the seventh day after transduction using flow cytometry.

Previously, we showed that the chimeric receptor Flt3-CAR was not cytotoxic towards Flt3-positive THP-1-mKate2 cells in case of deficient T-cell killing machinery [17]. Introducing mutations into a protein sequence may lead to changes in cytotoxicity. T-cell Jurkat cells were transduced with the same viruses to obtain a non-cytotoxic model of Flt3m-CAR T-cells. Using Western blotting with anti-CD3ζ chain staining, it was shown that Flt3m-CAR^EYFP−^ Jurkat cell and Flt3m-CAR Jurkat cell lysates contain chimeric receptors (Figure 4A, lanes 3 and 4, respectively). Transduction efficiency for Flt3m-CAR Jurkat and Flt3-CAR Jurkat cells was similar—44.1% and 43.1%, respectively.

**Figure 5:**
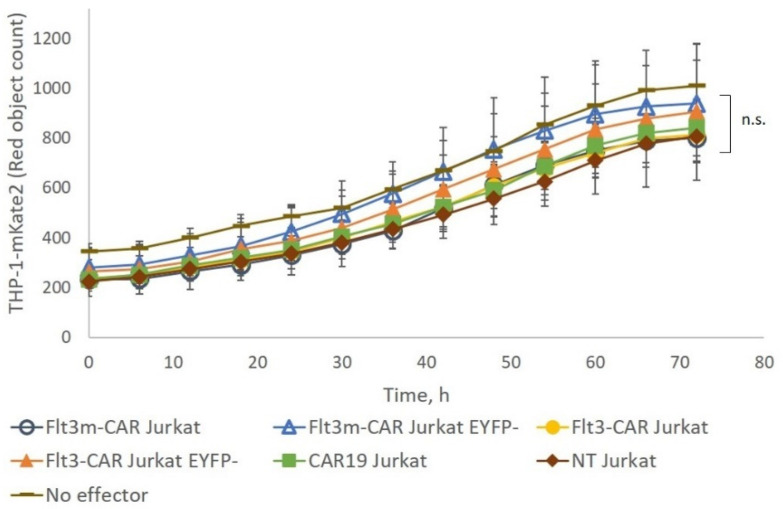
Flt3m-CAR Jurkat cells showed no cytotoxicity towards THP-1-mKate2 cells. Flt3m-CAR Jurkat cells, Flt3m-CAR^EYFP−^ Jurkat cells, Flt3-CAR Jurkat cells, Flt3-CAR^EYFP−^ Jurkat cells, CAR19 Jurkat cells, and non-transduced (NT) Jurkat cells were co-incubated with THP-1-mKate2 cells at an E:T ratio of 1:1. Empty dark blue circles refer to Flt3m-CAR T-cells, empty blue triangles to Flt3m-CAR^EYFP−^ T-cells, yellow circles to Flt3-CAR T-cells, orange triangles to Flt3-CAR^EYFP−^ T-cells, green squares to CAR19 T-cells, brown diamonds to NT T-cells, and dashes to wells with THP-1-mKate2 cells without any effector cells. Red object counts are shown in the figure, indicating the number of alive THP-1-mKate2 cells. Data are represented as mean ± SD. A Kruskal-Wallis test was performed to identify significant differences. n.s., not significant.

Flt3m-CAR Jurkat cells were incubated with Flt3-positive THP-1-mKate2 cells at an E:T ratio of 1:1 for 4 days (Figure 5). Flt3m-CAR Jurkat cells did not disrupt Flt3-positive cells during incubation, similar to Flt3-CAR Jurkat cells. Thus, we have confirmed that the L27P mutation in the Flt3Lg sequence in the Flt3m-CAR receptor did not make the receptor cytotoxic.

### 2.5. Recombinant Flt3Lg-L27P Did Not Interfere with Flt3m-CAR T-Cell-Mediated Killing

Previously, we demonstrated that soluble Flt3Lg dose-dependently inhibited wild-type Flt3Lg-based Flt3-CAR T-cell-mediated killing of Flt3-positive THP-1-mKate2 cells. To prove that the L27P mutation in the Flt3m-CAR recognizing domain sequence did not change the chimeric receptor’s recognition of Flt3, recombinant wild-type Flt3Lg was added to the co-incubation media at several concentrations. Flt3m-CAR T-cell-mediated cytotoxicity towards THP-1-mKate2 cells was dose-dependently inhibited by the soluble wild-type Flt3Lg, similar to what was observed in Flt3-CAR T-cells (Figure S2). Flt3m-CAR T-cells competed with the wild-type Flt3Lg for Flt3 binding sites.

Metalloproteinases of the ADAM family are known to cleave Flt3Lg from the membrane-bound form, making it soluble. Although particular cleavage sites are not yet known [21], it cannot be ruled out that the cleavage site may be present on the Flt3m-CAR sequence.

**Figure 6:**
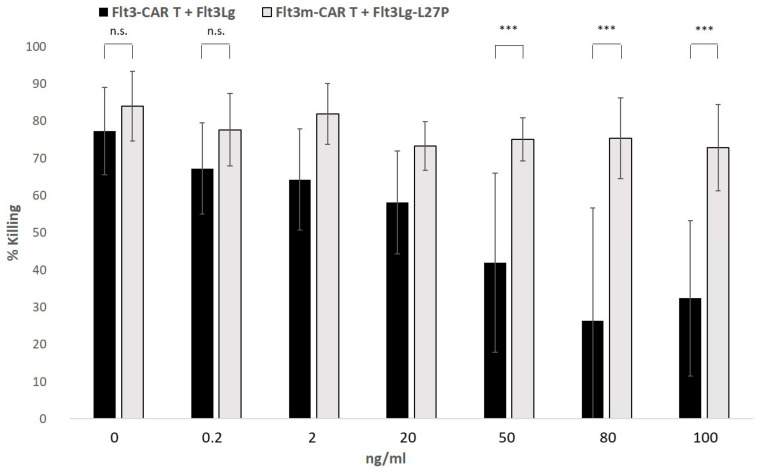
Flt3m-CAR T-mediated killing was not interfered with by recombinant Flt3Lg-L27P. Soluble Flt3Lg-L27P or Flt3Lg (0.2 ng/mL, 2 ng/mL, 20 ng/mL, or 100 ng/mL) was added to co-culture medium prior to the experiment. THP-1-mKate2 cells were incubated with Flt3m-CAR T-cells (light-grey bars) or Flt3-CAR T-cells (black bars). The relative number of alive THP-1-mKate2 cells after 45 h of co-incubation was used to define the % killing shown in the figure (Appendix A for details). Mann–Whitney U-tests were performed to identify significant differences. *** *p* < 0.001; n.s., not significant.

To test if soluble Flt3Lg-L27P would disrupt the cytotoxicity of Flt3m-CAR T-cells, Flt3-positive THP-1-mKate2 cells were incubated with Flt3m-CAR T-cells or Flt3-CAR T-cells at an E:T ratio of 1:1 in the presence of recombinant Flt3Lg-L27P or Flt3Lg for 45 h (Figure 6). A maximum concentration of 100 ng/mL of Flt3Lg-L27P was chosen because it corresponded to a higher plateau of proliferative activity for Flt3Lg-L27P.

Adding recombinant Flt3Lg-L27P to all the studied concentrations did not affect the cytotoxicity of Flt3m-CAR T-cells towards Flt3-positive THP-1-mKate2 cells.

### 2.6. Excess of Flt3m-CAR Jurkat Cells Could Not Interfere in Flt3m-CAR T-Cell-Mediated Cytotoxicity 

Flt3m-CAR T-cells and Flt3-CAR T-cells display full-length Flt3Lg-L27P or wild-type Flt3Lg as part of the chimeric receptor on their surface. Both cytokine variants are biologically active proteins. Presumably, the exhausted Flt3m-CAR T-cells and Flt3-CAR T-cells can interact with tumor cells through the Flt3-ligand:Flt3 receptor interface, blocking access to active Flt3m-CAR T-cells and Flt3-CAR T-cells. Alternatively, a membrane-bound form of Flt3Lg, which is expressed in a variety of cells including fibroblasts, can also interfere in the Flt3m-CAR T-cell-mediated killing.

**Figure 7:**
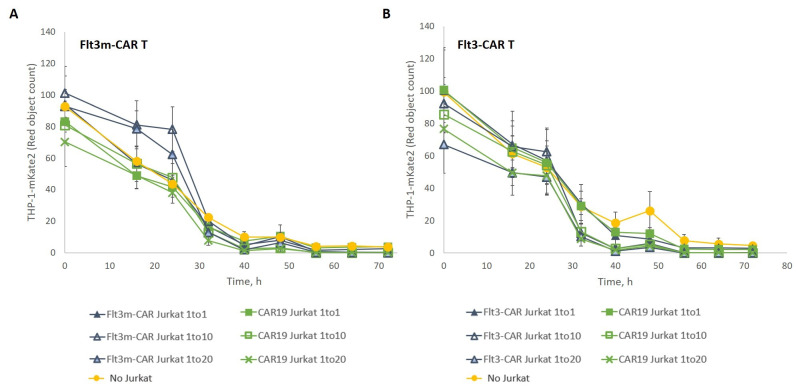
An excess of Flt3m-CAR Jurkat cells or Flt3-CAR Jurkat cells did not disrupt Flt3m-CAR T-cell- or Flt3-CAR T-cell-mediated killing, respectively. THP-1-mKate2 cells were incubated with either Flt3m-CAR T-cells (**A**) or Flt3-CAR T-cells (**B**) (E:T = 1:1) in the presence of different amounts (1×; 10×, 20×) of Flt3m-CAR Jurkat cells or Flt3-CAR Jurkat cells, respectively. Triangles refer to Flt3m-CAR Jurkat (**A**) or Flt3-CAR Jurkat (**B**), with filled triangles referring to 1×, light blue triangles to 10× excess, and empty triangles to 20× excess. Green curves refer to CAR19 Jurkat cells (**A**,**B**), with filled squares referring to 1×, empty squares to 10× excess, and crosses to 20× excess. Yellow circles refer to control curves where no Jurkat cells were added (**A**,**B**). Data are represented as mean ± SD.

Flt3m-CAR T-cells and Flt3-CAR T-cells were incubated with Flt3-positive THP-1-mKate2 target cells at an E:T ratio of 1:1 in the presence of non-cytotoxic Flt3m-CAR Jurkat cells and Flt3-CAR Jurkat cells, respectively (Figure 7). Even the presence of a 20-fold excess of both non-cytotoxic Flt3m-CAR Jurkat cells and Flt3-CAR Jurkat cells did not affect the Flt3m-CAR T-cell- or Flt3-CAR T-cell-mediated killing of Flt3-positive THP-1-mKate2 cells.

## 3. Discussion

Determined by a particular scFv sequence, the off-target activation of CAR T-cells detrimentally affects the specificity and potency of CAR T-cell therapy in vivo. To decrease tonic activation, scFv sequence modifications may be required. In terms of scFv construct modifications, further research is needed to prove the thermostability or pH stability and functionality of the new scFv protein. Using natural ligand sequences in chimeric antigen receptors’ recognizing domain-coding regions may reduce the tonic activation of CAR T-cells and therefore delay their exhaustion. The widely known characteristics of ligands may allow for the precise tuning of recognition affinity. For example, introducing a point mutation to the sequence of the ligand-based extracellular part of the IL13Rα2-CAR receptor led to 50-fold higher affinity for the target molecule IL13Rα2 and 5-fold lower affinity for IL13Rα1 [16].

Among the benefits of natural ligand-based targeting, the high affinity of ligand–receptor binding can be considered. In the case of AML though, tumor-associated antigens are often normal proteins that are overexpressed. Lower-affinity CAR T-cells (µM) required higher concentrations of the target antigen to activate than higher-affinity CAR T-cells (nM) [22]. Moreover, low-affinity CAR T-cells were cytotoxic towards the cells overexpressing the target antigen, but not to the cells with a lesser amount of the target antigen. For instance, the E21 position in the GM-CSF sequence is required for the high-affinity ligand–receptor complex formation necessary for intracellular signaling [23]. The E21K mutation was introduced into the GM-CSF sequence in the GMR-CAR construct. GMR-CAR T-cells eliminated normal monocytes, but not the normal neutrophils that bear lower amounts of the GM-CSFRα chain [24].

Various mutant Flt3Lg proteins were tested previously [18]. The authors found that the L27P point mutation in the Flt3Lg sequence significantly reduced the specific activity of Flt3Lg, while ligand–receptor binding was preserved. In 2021, the X-ray crystallographic structure of Flt3Lg-L27D was published [19]. According to the revealed structural properties, it is the 3D structure of the Flt3Lg dimer that is most affected by mutation. In this work, we purified the recombinant Flt3Lg-L27P produced in a mammalian system. We confirm that the ED_50_ of Flt3Lg-L27P was at least 10-fold higher than that of wild-type Flt3Lg.

We present ligand-based Flt3m-CAR T-cells bearing the L27P mutation in the Flt3Lg sequence of the recognizing domain. Flt3m-CAR T-cells retain the cytotoxic properties and specificity of ligand-based Flt3-CAR T-cells.

In a paper devoted to the use of ligand-based TPO-CAR T-cells against the MPL receptor, the authors proposed thrombopoietin shedding from the surface of TPO-CAR T-cells [25]. Stem cell factor (SCF), which belongs to the same cytokine family as Flt3Lg, has two main isoforms—soluble and membrane-bound [26]. For SCF, the cleavage site was found. SCF may lose the cleavage site as a result of alternative splicing and become membrane bound. Otherwise, SCF may be cleaved from the cellular surface resulting in soluble SCF. Both membrane-bound and soluble forms were also described for Flt3Lg. Although the particular cleavage site for Flt3Lg is not yet known, ADAM family metalloproteinases are revealed to cleave Flt3Lg from the cell surface [21]. It cannot be ruled out that full-length Flt3Lg may be cleaved from Flt3-CAR T-cells and become part and parcel of the systemic circulation.

Wild-type Flt3Lg is present in the plasma of healthy individuals at low concentrations (~1 pg/mL). The concentration level of soluble Flt3Lg may increase in the case of AML by up to ~20 ng/mL during induction chemotherapy [27]. The authors associated the increase in Flt3Lg levels in plasma with good prognosis. The issue of whether the elevated Flt3Lg plasma level is a direct outcome of the reduced tumor mass and the reduced amount of Flt3Lg consumed by blast cells or Flt3Lg playing some crucial yet unknown function during therapy is of considerable importance but lies beyond of the scope of this work.

In this work, we confirmed that soluble recombinant Flt3Lg induces downregulation of the Flt3 receptor on THP-1 cells, as was described previously [20]. At the same time, we found that concentrations as high as 2 ng/mL of soluble recombinant Flt3Lg-L27P did not reduce the amount of Flt3 receptors of the THP-1 cell surface. Therefore, we may propose that an increase in circulating soluble Flt3Lg-L27P in the case of cleavage from Flt3m-CAR T-cells should not participate in possible Flt3-negative relapse.

We found that soluble Flt3Lg-L27P in concentrations of up to 100 ng/mL did not disrupt the elimination of Flt3-positive THP-1-mKate2 cells by Flt3m-CAR T-cells. Additionally, we showed that up to 2 ng/mL of soluble Flt3Lg-L27P has little effect on the ED_50_ of wild-type Flt3Lg. Thus, we propose that in the case of Flt3Lg-L27P cleavage from Flt3m-CAR T-cells, soluble Flt3Lg-L27P should not disrupt the natural functions of endogenous Flt3Lg in the systemic circulation.

To summarize, ligand-based Flt3m-CAR T-cells retain the specificity of ligand-based Flt3-CAR T-cells. Flt3m-CAR T-cells containing the Flt3Lg-L27P mutated sequence in the recognizing part may be safer than Flt3-CAR T-cells due to the reduced bioactivity of Flt3Lg-L27P. Further in vivo testing is required to verify the approach.

## 4. Materials and Methods

### 4.1. Flt3Lg-L27P Cloning and Purification

Flt3Lg-L27P mutant cDNA was generated from wild-type Flt3Lg cDNA by PCR with Q5 polymerase (NEB). The primers used for mutagenesis were ForwardL27P actacctgcctcaagattacccagtcaccg and ReverseL27P ttgaggcaggtagtcagacagctc. Final cDNA with an introduced DED epitope in the C-terminal part of the Flt3Lg-L27P mutant was cloned into an expression vector and was generated by selective cloning after transfection of a stable clone of CHO cells. CHO cells were cultivated in OPTI-CHO medium (‘Gibco’, Billings, MT, USA) at a density of about 10^6^ cells per mL at 37 °C and 7% CO_2_ with shaking. The supernatant of CHO cells was used for affinity purification of Flt3Lg-L27P protein with an anti-DED affinity column. The purified protein was transferred to PBS solution by diafiltration, sterile filtered, and stored at −80 °C.

### 4.2. CAR Lentiviruses Generation

Flt3-CAR- and CAR19-coding lentiviruses were designed as described previously [17]. The packaging cell line HEK293T was transfected with the plasmids, and the lentiviral particles were harvested 48 h after transfection and concentrated. The aliquots of concentrated viruses were stored at −70 °C until use.

The NotI site was introduced after the Flt3Lg-L27P mutant sequence by PCR and cloned to be in frame with the CAR sequence, used formerly for wild-type Flt3-CAR [17]. Final cDNA was transferred to a pLVX-EF1alfa vector (‘Takara’, Kusatsu, Japan).

### 4.3. Cell Culture

All cells were cultured in a humidified atmosphere of 5% CO_2_ at 37 °C.

Human HEK293T, Jurkat, THP-1, and U937 cells were obtained from the Russian Cell Culture Collection of Vertebrate Cells (Institute of Cytology, Russian Academy of Sciences, St. Petersburg, Russia).

HEK293T cells were cultured in DMEM medium (‘Gibco’, Billings, MT, USA) with the addition of 10% fetal bovine serum (‘Gibco’, Billings, MT, USA), 2 mM L-Glutamine (‘HyClone’, Logan, UT, USA), 100 U/mL penicillin, and 100 µg/mL streptomycin.

THP-1, THP-1-mKate2, U937, U937-mKate2, Jurkat, and CAR-Jurkat cells were cultured in RPMI medium (‘Gibco’, Billings, MT, USA) with the addition of 10% fetal bovine serum (‘Gibco’, Billings, MT, USA) and 2 mM L-Glutamine (‘HyClone’, Logan, UT, USA).

Native and transduced T-cells were cultured in TexMACS (‘Miltenyi Biotec’, Bergisch Gladbach, Germany) medium with the addition of IL-7 and IL-15 (25 ng/mL, ‘SCI-Store’, Moscow, Russia).

### 4.4. Flt3m-CAR T-Cell and CAR Jurkat Cell Generation

Apheresis products were used to obtain donor T-cells using CliniMACS Prodigy equipment (‘Miltenyi Biotec’, Bergisch Gladbach, Germany). T-cells were seeded at a density of 10^6^ cells/well in a 24-well plate, activated with TransAct reagent (‘Miltenyi Biotec’, Bergisch Gladbach, Germany), and transduced. Jurkat cells were seeded at a density 5 × 10^5^ cells/well in a 24-well plate and transduced.

Transduction efficiency was estimated using flow cytometry. EYFP-positive cells were referred to as CAR-positive cells.

### 4.5. Western-Blotting

Transduced T-cells and Jurkat cells were washed twice in DPBS (‘HyClone’, Logan, UT, USA), lysed in Laemmli buffer with the addition of β-mercaptoethanol (‘Bio-Rad’, Hercules, CA, USA), and incubated for 10 min at 100 °C. Proteins were resolved using SDS-PAGE techniques and then shifted to a PVDF membrane (‘GE Healthcare’, Chicago, IL, USA). The blocking of non-specific interactions was performed using 1% ECL Block (‘GE Healthcare’, Chicago, IL, USA). Proteins were stained with anti-CD3ζ chain (‘Sigma’, St. Louis, MO, USA), anti-GAPDH (‘Cell Signaling Technology’, Danvers, MA, USA), and anti-EYFP (‘SCI-Store’, Moscow, Russia) antibodies.

Chemiluminescence was detected after incubation of the membrane with anti-rabbit-HRP antibodies (for anti-CD3ζ chain and anti-GAPDH; ‘GE Healthcare’, Chicago, IL, USA) or anti-mouse-IgG-HRP antibodies (for anti-EYFP; ‘GE Healthcare’, Chicago, IL, USA).

### 4.6. Flow Cytometry

All flow cytometry experiments were performed using a Navios flow cytometer (‘Beckman Coulter’, Brea, CA, USA).

THP-1 cells were incubated with recombinant Flt3Lg or Flt3Lg-L27P if needed. THP-1 cells and U937 cells were washed in DPBS (‘HyClone’, Logan, UT, USA), incubated with anti-CD135 PE (‘Beckman Coulter’, Brea, CA, USA) for 15 min at room temperature, and then washed and counted.

CAR T-cells, non-transduced T-cells, CAR Jurkat cells, and Jurkat cells were washed in DPBS (‘HyClone’, Logan, UT, USA) and counted.

### 4.7. CAR T-Cell and CAR Jurkat Cell Cytotoxicity Assay

CAR T-cells were transferred to IL-7- and IL-15-deficient TexMACS medium (‘Miltenyi Biotec’, Bergisch Gladbach, Germany) and incubated for 3 d until rested.

A 96-well plate was pre-coated with 10 µg/mL of human plasma fibronectin (‘IMTEK’, Moscow, Russia) for 1 h at room temperature. The wells were washed with DPBS (‘HyClone’, Logan, UT, USA). CAR T-cells were transferred to RPMI complete medium. CAR T-cells were mixed with THP-1-mKate2 cells and U937-mKate cells at an E:T ratio of 1:1 or 5:1 and incubated for 4 d (37 °C, 5% CO_2_). The number of red objects (mKate2-fluorescent target cells) was analyzed every 4 h using an IncuCyte S3 Live Imaging System (‘Sartorius’, Goettingen, Germany).

CAR Jurkat cells were mixed with THP-1-mKate2 cells at an E:T ratio of 1:1, 10:1, or 20:1, incubated, and analyzed similarly.

Soluble recombinant Flt3Lg (‘SCI-Store’, Moscow, Russia) and Flt3Lg-L27P were added to the co-culture medium before cell suspension mixing to the final concentrations of 0.2 ng/mL, 2 ng/mL, 20 ng/mL, and 100 ng/mL.

### 4.8. Cytokine Secretion

To measure cytokine secretion, 10^4^ Flt3-CAR^EYFP−^ T-cells were incubated with THP-1, U937, or without target cells (E:T = 1:1) for 24 h. After incubation, the cell suspensions were centrifuged, and the supernatants were aliquoted and frozen until use (−80 °C). IFNγ and TNFα were measured using ELISA kits (‘Vektor-Best’, Novosibirsk, Russia) in accordance with the manufacturer’s protocol. The experiments were performed twice.

### 4.9. Proliferation Assay

To compare the bioactivity of soluble recombinant Flt3Lg (‘SCI-Store’, Moscow, Russia) and Flt3Lg-L27P, the THP-1 cell line was used. Soluble Flt3Lg and Flt3Lg-L27P were diluted threefold starting from 30 ng/mL in a 96-well plate. Concentrations of 0.1 ng/mL or 2 ng/mL of recombinant Flt3Lg-L27P were added to the Flt3Lg-containing culture medium when needed.

THP-1 cells were washed in a low-serum medium and added to the plates. THP-1 cells were incubated with either recombinant Flt3Lg or Flt3Lg-L27P for 2 d (37 °C, 5% CO_2_). Stimulated proliferation was assessed using the CellTiter 96® AQueous One Solution assay (‘Promega’, Madison, WI, USA) in accordance with the manufacturer’s protocol.

### 4.10. Statistical Analysis

Flow cytometry data were analyzed using Kaluza software. For proliferative tests, all concentrations of proteins were assessed in triplicate, and the data were represented as mean ± standard deviation. For cytotoxicity assays, the data were analyzed using Mann–Whitney U-tests and Kruskal–Wallis tests. Nine locations were captured in each well. Experiments were performed at least three times.

## Data Availability

The raw data supporting the conclusions of this article are available on request without undue reservation.

## Supplementary Materials

The supporting information can be downloaded at: https://www.mdpi.com/article/10.3390/ijms24087626/s1.

## Appendix A

To determine ‘% Killing’ (Figure 6), the mean number of living THP-1-mKate2 cells (#targets) after 45 h of co-incubation was normalized to that of the beginning of the experiment for Flt3m-CAR T-cells, Flt3-CAR T-cells, and NT T-cells for each Flt3Lg or Flt3Lg-L27P concentration tested. The normalized #targets for Flt3-CAR T-cells and for Flt3m-CAR T-cells were then divided by #targets for NT T-cells, representing the ‘% Lysis’. The ‘% Killing’ was equal to ‘100—% Lysis’.
