# Supplementary figures and images for: Mutated Flt3Lg Provides Reduced Flt3 Recycling Compared to Wild-Type Flt3Lg and Retains the Specificity of Flt3Lg-Based CAR T-Cell Targeting in AML Models
Source: Int J Mol Sci. 2023 Apr 21;24(8):7626. doi: 10.3390/ijms24087626 (PMC10146938; doi:10.3390/ijms24087626)

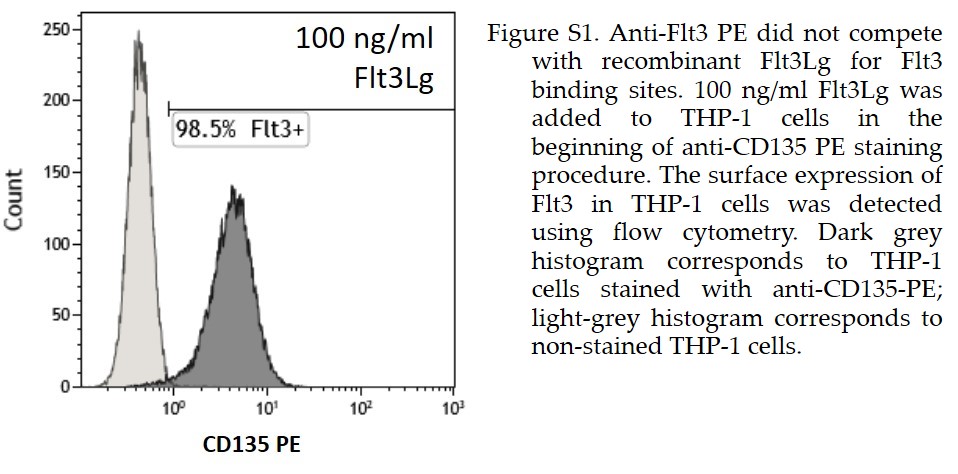

Supplement: Supplementary file 1 [file ijms-24-07626-s001.zip › Figure S1.jpg]

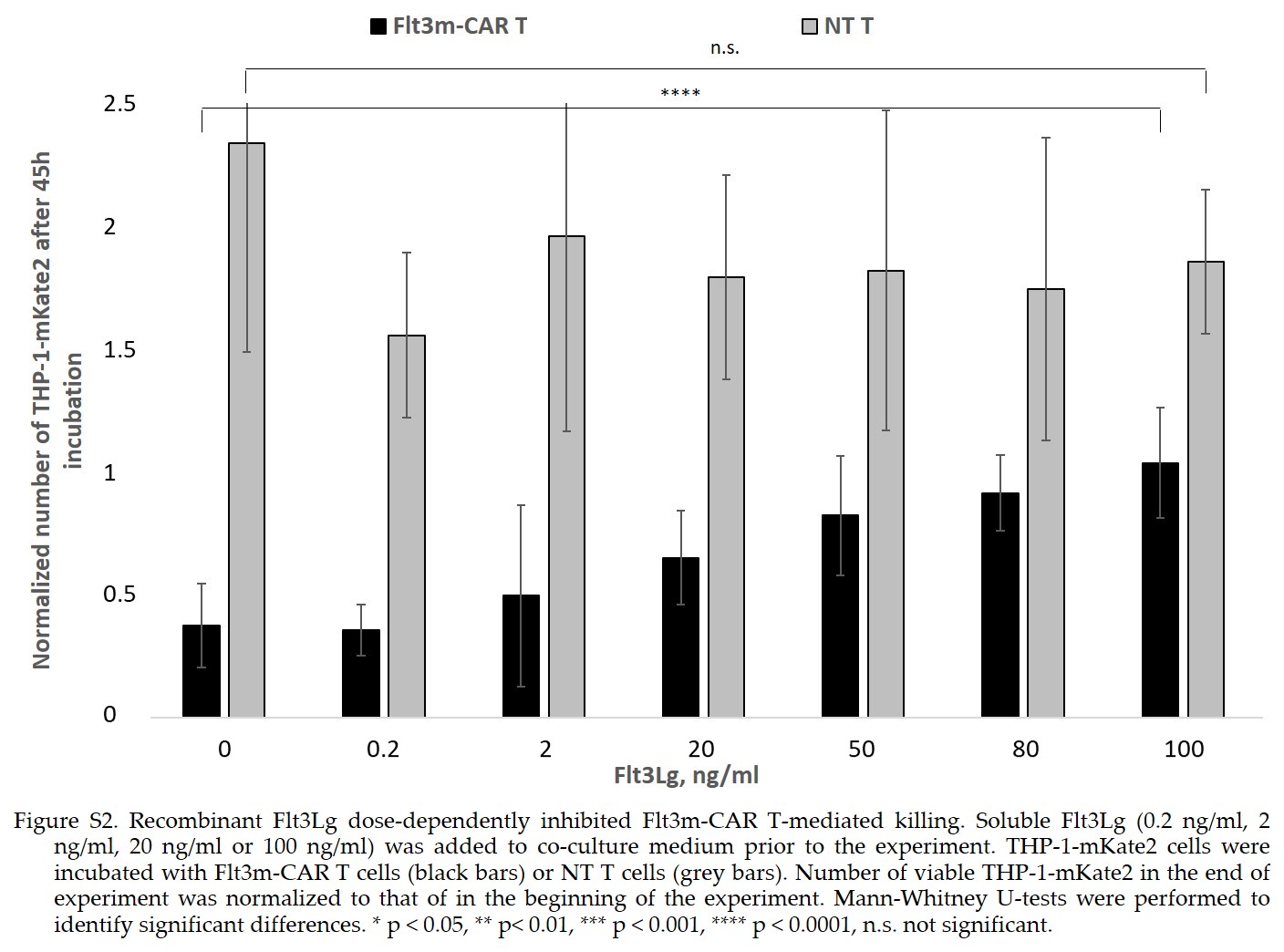

Supplement: Supplementary file 1 [file ijms-24-07626-s001.zip › Figure S2.jpg]

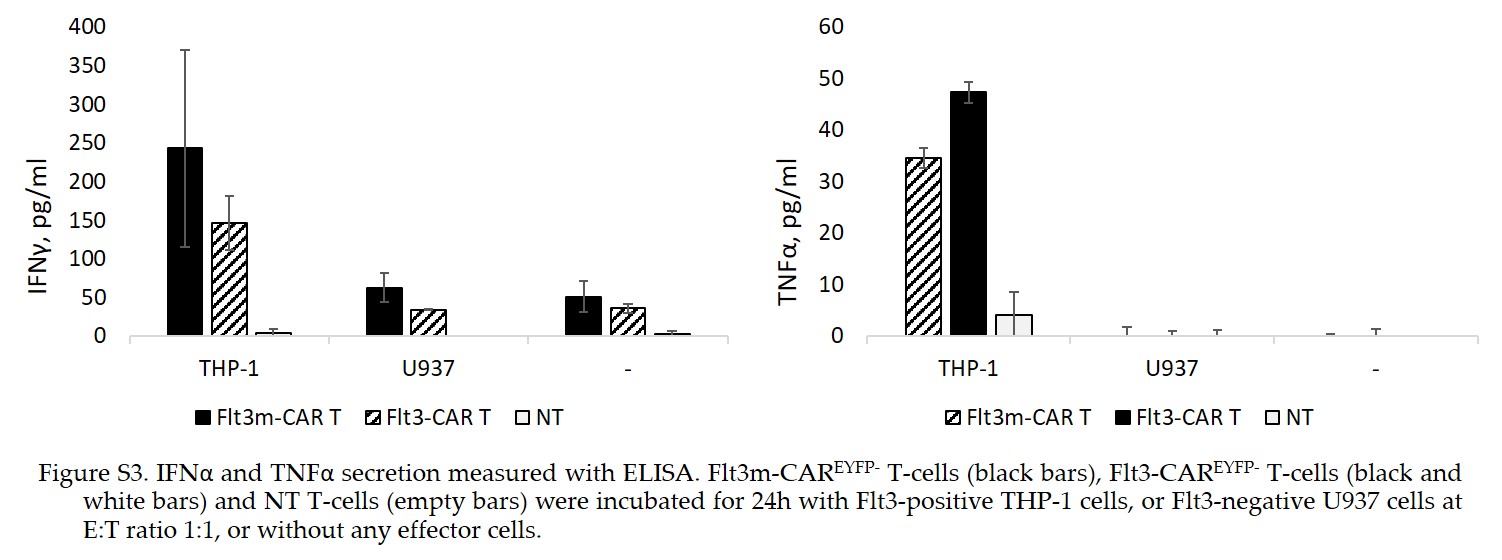

Supplement: Supplementary file 1 [file ijms-24-07626-s001.zip › Figure S3.jpg]
